# Supplementary material for: BakRep – a searchable large-scale web repository for bacterial genomes, characterizations and metadata
Source: Microb Genom. 2024 Oct 30;10(10):001305. doi: 10.1099/mgen.0.001305 (PMC11524574; doi:10.1099/mgen.0.001305)
Supplement: Uncited Supplementary Material 1. [file mgen-10-01305-s001.pdf]

# Supplemental Data

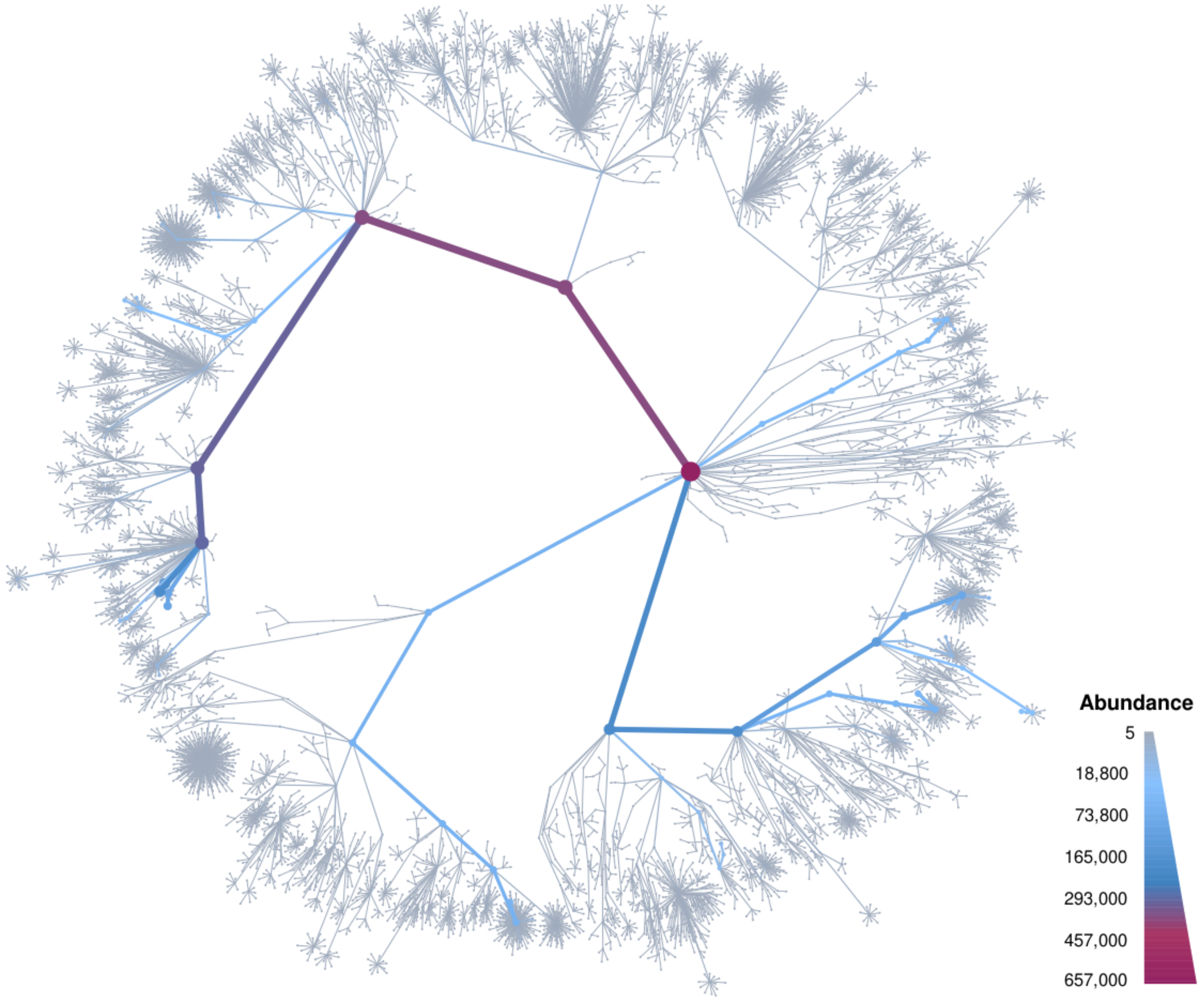


**Supplemental Figure 1:** Overview of the taxonomic composition at the species level. Nodes and branches are colored and sized by aggregated genome abundances at each taxonomic rank. The figure was created using the Metacoder package.

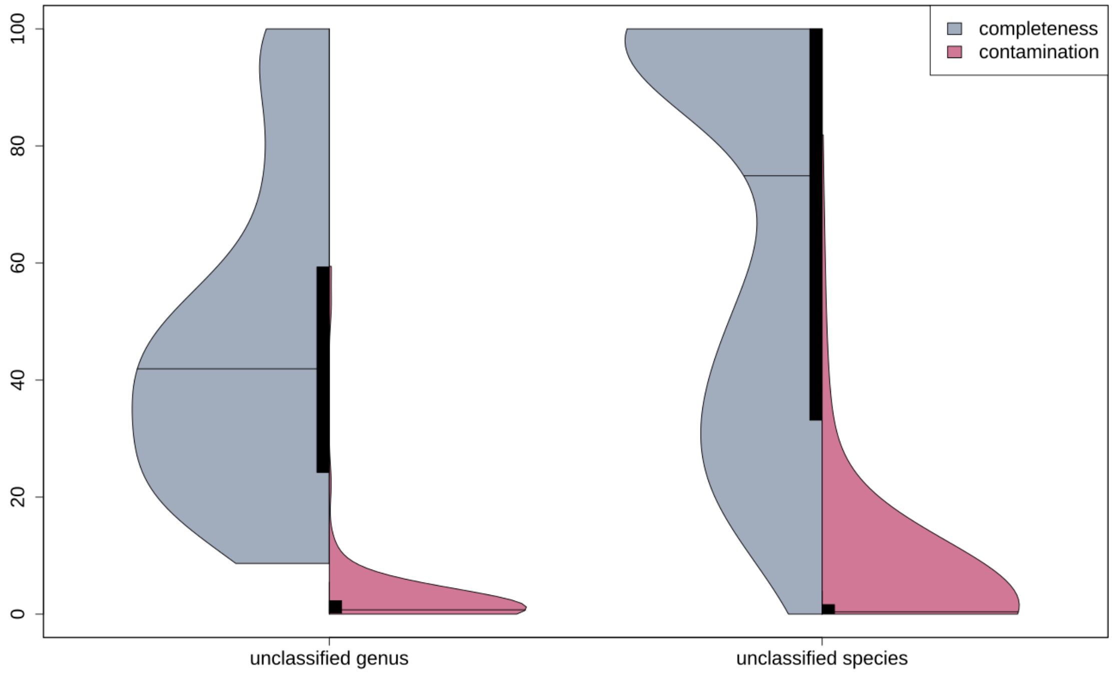


**Supplemental Figure 2:** Distribution of completeness and contamination level of all unclassified genomes. Completeness is displayed on the left of each plot (gray), and contamination is displayed on the right (magenta). Plots are created with R version 4.3.1.

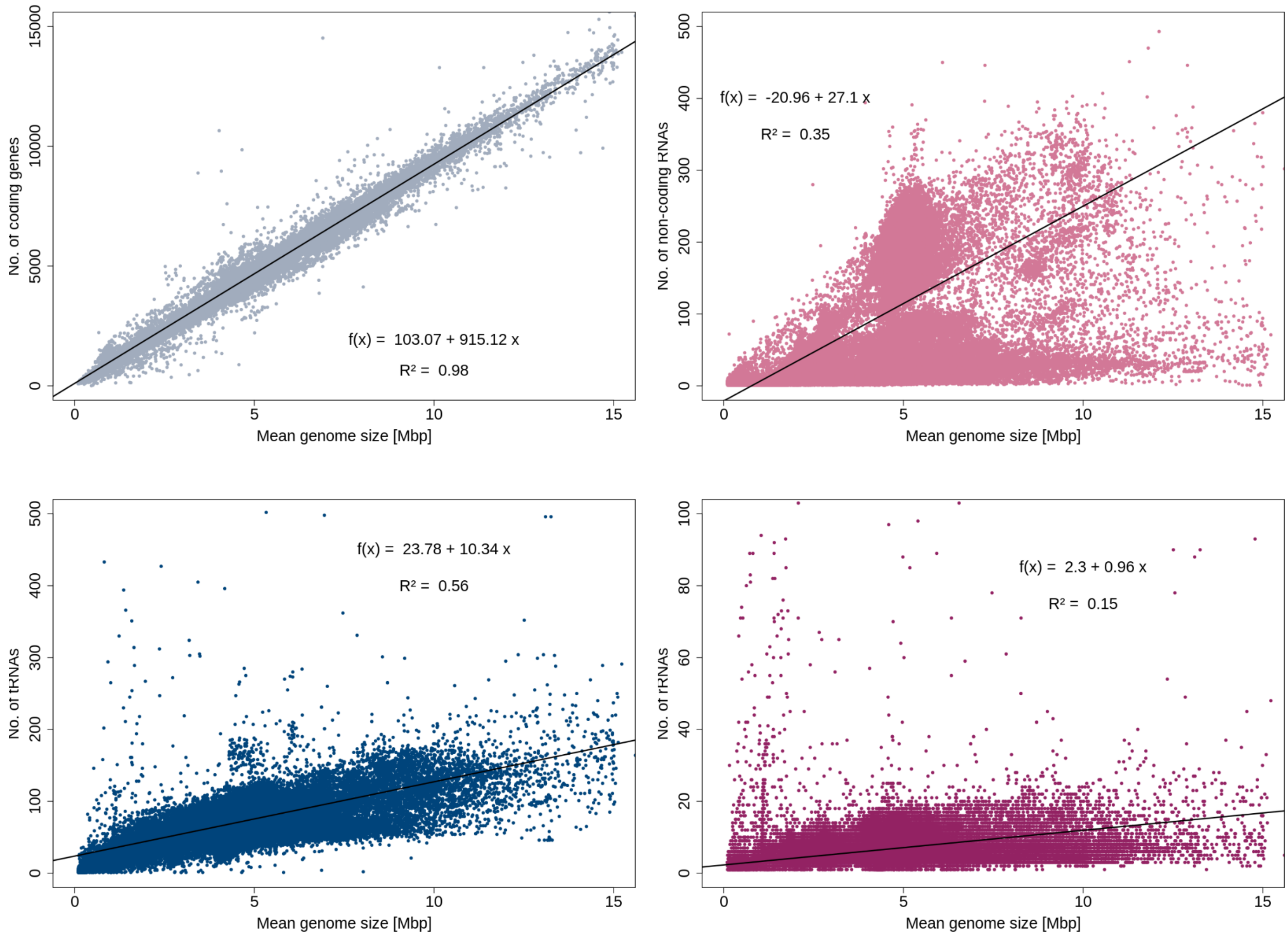


**Supplemental Figure 3:** Distribution of annotation feature counts. The mean number of coding genes (top left), non-coding RNAs (top right), tRNAs (bottom left), and rRNAs (bottom right) is shown against the mean genome size per species. A linear fit is drawn to emphasize if the respective distribution follows a linear trend. Plots are created with R version 4.3.1.
